# (*E*)-7-(Pyren-1-yl)hept-6-enoic acid

**DOI:** 10.1107/S1600536810024499

**Published:** 2010-06-26

**Authors:** Arto Valkonen, Tanja Lahtinen, Kari Rissanen

**Affiliations:** a Department of Chemistry, Nanoscience Center, University of Jyväskylä, PO Box 35, FIN-40014 University of Jyväskylä, Finland

## Abstract

The title compound, C_23_H_20_O_2_, is a precursor of a pyrene-based supra­molecular element for non-covalent attachment to a carbon nanotube. The asymmetric unit contains three independent mol­ecules. The carb­oxy­lic acid group in each of these mol­ecules serves as an inter­molecular hydrogen-bond donor and acceptor, generating the commonly observed double O—H⋯O hydrogen-bond motif in an eight-membered ring. Weaker C—H⋯O, π–π [centroid–centroid distance = 3.968 (4) Å] and C—H⋯π inter­actions are also found in the crystal structure.

## Related literature

Pyrene functionalized with an aliphatic spacer can be used to functionalize mol­ecular skeletons and the resulting mono- or multipyrene derivative bound non-covalently to a π-surface, see: Kavakka *et al.* (2007 ▶); Tomonari *et al.* (2006 ▶). For related structures, see: Bariamis *et al.* (2009 ▶). For hydrogen-bond motifs, see: Bernstein *et al.* (1995 ▶). The title compound was synthesized by a Wittig reaction, see: Wittig & Haag (1955 ▶); Wittig & Schöllkopf (1954 ▶).
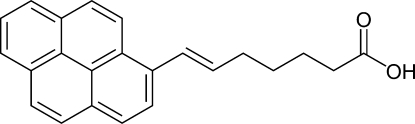


## Experimental

### 

#### Crystal data

C_23_H_20_O_2_
*M* *_r_* = 328.39
Triclinic, 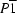

*a* = 10.7785 (3) Å
*b* = 13.3315 (4) Å
*c* = 18.9574 (6) Å
α = 103.287 (2)°
β = 103.064 (2)°
γ = 98.391 (2)°
*V* = 2525.64 (13) Å^3^
*Z* = 6
Mo *K*α radiation
μ = 0.08 mm^−1^
*T* = 123 K
0.32 × 0.14 × 0.06 mm

#### Data collection

Bruker–Nonius Kappa CCD diffractometer with an APEXII detector
15113 measured reflections
8885 independent reflections
5729 reflections with *I* > 2σ(*I*)
*R*_int_ = 0.038

#### Refinement

*R*[*F* ^2^ > 2σ(*F* ^2^)] = 0.064
*wR*(*F* ^2^) = 0.164
*S* = 1.06
8885 reflections
685 parameters
9 restraints
H atoms treated by a mixture of independent and constrained refinement
Δρ_max_ = 0.32 e Å^−3^
Δρ_min_ = −0.39 e Å^−3^

### 

Data collection: *COLLECT* (Bruker, 2008 ▶); cell refinement: *DENZO-SMN* (Otwinowski & Minor, 1997 ▶); data reduction: *DENZO-SMN*; program(s) used to solve structure: *SIR2004* (Burla *et al.*, 2005 ▶); program(s) used to refine structure: *SHELXL97* (Sheldrick, 2008 ▶); molecular graphics: *ORTEP-3* (Farrugia, 1997 ▶) and *Mercury* (Macrae *et al.*, 2008 ▶); software used to prepare material for publication: *SHELXL97*.

## Supplementary Material

Crystal structure: contains datablocks global, I. DOI: 10.1107/S1600536810024499/fj2323sup1.cif

Structure factors: contains datablocks I. DOI: 10.1107/S1600536810024499/fj2323Isup2.hkl

Additional supplementary materials:  crystallographic information; 3D view; checkCIF report

## Figures and Tables

**Table 1:** Hydrogen-bond geometry (Å, °) *Cg*1 and *Cg*2 are the centroids of the C1*A*–C4*A*,C15*A*,C14*A* and C7*C*–C11*C*,C16*C* rings, respectively.

*D*—H⋯*A*	*D*—H	H⋯*A*	*D*⋯*A*	*D*—H⋯*A*
O2*A*—H2*O*⋯O1*C*^i^	0.88 (2)	1.79 (2)	2.661 (3)	177 (3)
O2*B*—H2*P*⋯O1*B*^ii^	0.87 (2)	1.78 (2)	2.642 (3)	173 (3)
O2*C*—H2*Q*⋯O1*A*^iii^	0.86 (2)	1.78 (2)	2.636 (3)	172 (3)
C10*C*—H10*C*⋯O1*A*^iv^	0.95	2.46	3.336 (3)	154
C10*B*—H10*B*⋯O1*B*^v^	0.95	2.51	3.354 (3)	148
C10*A*—H10*A*⋯O1*C*^vi^	0.95	2.51	3.360 (3)	150
C22*C*—H22*E*⋯*Cg*2^vii^	0.99	2.63	3.500 (4)	147
C19*A*—H19*A*⋯*Cg*1^viii^	0.99	2.72	3.511 (4)	137

Symmetry codes: (i) 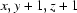; (ii) 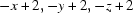; (iii) 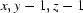; (iv) 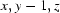; (v) 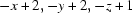; (vi) 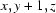; (vii) 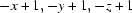; (viii) 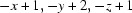.

**Fig. 1.**
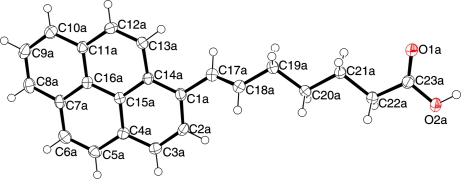
View of the molecule A of title compound showing the atom-labeling scheme. Displacement ellipsoids are drawn at the 50% probability level. H atoms are represented by circles of arbitrary size.

**Fig. 2.**
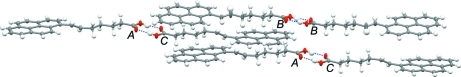
Packing diagram of the title compound showing the AC and BB pairs formed by O—H···O interactions between carboxyl groups.

## supplementary crystallographic information

### Comment

Pyrene functionalized with an aliphatic spacer can be used to functionalize molecular skeletons and the resulting mono- or multipyrene derivative bound non-covalently to a pi-surface. Such a non-covalent functionalization has recently been reported by Kavakka *et al.* (Kavakka, *et al.*, 2007) and Tomonari *et al.* (Tomonari, *et al.*, 2006).

The title compound is synthesized by a Wittig reaction (Wittig & Schöllkopf, 1954; Wittig & Haag, 1955). In the molecule (Fig. 1) the planar pyrene ring is bonded to olefinic end carbon of hepten-6-oic acid chain and the pyrene is not coplanar with the conjugated double bond. In three independent molecules in asymmetric unit (labelled *A*, *B* and *C*) the hepten-6-oic acid side chains are almost straight and the dihedral angles between the pyrene rings and the double bonds are 34.8 (3), 14.5 (5) and 33.7 (2) °, respectively. Similar phenomenon was observed with (2*E*,4*E*,6*E*)-3-Methyl-7-(pyren-1-yl)octa-2,4,6-trienoic acid (Bariamis, *et al.*, 2009), the trienoic acid derivative with two additional methyl groups in the positions of 3 and 7 in the side chain. Similar to that trienoic acid (Bariamis, *et al.*, 2009) the carboxyl groups of three molecules of the title compound are connected to carboxyl group of another molecule with double hydrogen bonding interactions (Table 1), generating the *R*^2^_2_(8) graph-set motif (Bernstein *et al.*, 1995). With this motif one centrosymmetric *BB* pair and two non-centrosymmetric *AC* pairs were formed (Fig. 2). Mercury -program (Macrae *et al.*, 2008) also finds some short intermolecular C—H···O, π–π [centroid-centroid distance 3.968 (4) Å] and weak C—H···π contacts (Table 1), which connect these pairs to each other and define the overall structure in the crystal.

### Experimental

Bis(trimethylsilyl)amine (HMDS) (526 mg, 3.28 mmol) was dissolved in dry THF under inert atmosphere. 1.6-*M* n-BuLi (6.15 ml) was added at -10 °C temperature and the resulting solution was placed into dropping funnel and kept under inert atmosphere. (5-Carboxypentyl)triphenylphosphonium bromide (1.5 g, 3.28 mmol) was dissolved in dry THF under inert atmosphere. The solution was cooled to -10 °C and LiHMDS was added dropwise to the reaction flask. After addition the reaction mixture was stirred at room temperature for 20 minutes. Then the reaction mixture was cooled again (ice salt bath) and 1-pyrene carboxaldehyde (755 mg, 3.28 mmol), dissolved in dry THF, was added dropwise into reaction mixture under inert atmosphere. After addition the reaction mixture was stirred at room temperature for 1 h. The reaction was quenched with aq. 5% citric acid, extracted with DCM and obtained extract was evaporated to yield brown oil. Brown plates were crystallized out from this oil within two weeks. These plates were directly used in single-crystal analysis.

### Refinement

All H atoms were visible in electron density maps, but those bonded to C were calculated at their idealized positions and allowed to ride on their parent atoms at C—H distances of 0.95 Å (aromatic, olefinic) and 0.99 Å (methylene), with *U*_iso_(H) of 1.2 times *U*_eq_(C). The O—H protons were found in the electron density map and were fixed in place by *DFIX* restraint (s = 0.02) at distances of 0.91 Å from N atoms, and *U*_iso_(H) values of 1.5 times *U*_eq_(O) were used.

### Crystal data

**Table:**

C_23_H_20_O_2_	*Z* = 6
*M**_r_* = 328.39	*F*(000) = 1044
Triclinic, *P*1	*D*_x_ = 1.295 Mg m^−^^3^
Hall symbol: -P 1	Mo *K*α radiation, λ = 0.71073 Å
*a* = 10.7785 (3) Å	Cell parameters from 10136 reflections
*b* = 13.3315 (4) Å	θ = 0.4–28.3°
*c* = 18.9574 (6) Å	µ = 0.08 mm^−^^1^
α = 103.287 (2)°	*T* = 123 K
β = 103.064 (2)°	Plate, brown
γ = 98.391 (2)°	0.32 × 0.14 × 0.06 mm
*V* = 2525.64 (13) Å^3^	

### Data collection

**Table:**

Bruker–Nonius Kappa CCD diffractometer with an APEXII detector	5729 reflections with *I* > 2σ(*I*)
Radiation source: fine-focus sealed tube	*R*_int_ = 0.038
graphite	θ_max_ = 25.0°, θ_min_ = 2.0°
Detector resolution: 9 pixels mm^-1^	*h* = −12→12
φ and ω scans	*k* = −15→14
15113 measured reflections	*l* = −22→22
8885 independent reflections	

### Refinement

**Table:**

Refinement on *F*^2^	Primary atom site location: structure-invariant direct methods
Least-squares matrix: full	Secondary atom site location: difference Fourier map
*R*[*F*^2^ > 2σ(*F*^2^)] = 0.064	Hydrogen site location: inferred from neighbouring sites
*wR*(*F*^2^) = 0.164	H atoms treated by a mixture of independent and constrained refinement
*S* = 1.06	*w* = 1/[σ^2^(*F*_o_^2^) + (0.051*P*)^2^ + 2.3851*P*] where *P* = (*F*_o_^2^ + 2*F*_c_^2^)/3
8885 reflections	(Δ/σ)_max_ < 0.001
685 parameters	Δρ_max_ = 0.32 e Å^−^^3^
9 restraints	Δρ_min_ = −0.39 e Å^−^^3^

### Special details

**Table:**

Geometry. All s.u.'s (except the s.u. in the dihedral angle between two l.s. planes) are estimated using the full covariance matrix. The cell s.u.'s are taken into account individually in the estimation of s.u.'s in distances, angles and torsion angles; correlations between s.u.'s in cell parameters are only used when they are defined by crystal symmetry. An approximate (isotropic) treatment of cell s.u.'s is used for estimating s.u.'s involving l.s. planes.
Refinement. Refinement of *F*^2^ against ALL reflections. The weighted *R*-factor *wR* and goodness of fit *S* are based on *F*^2^, conventional *R*-factors *R* are based on *F*, with *F* set to zero for negative *F*^2^. The threshold expression of *F*^2^ > 2σ(*F*^2^) is used only for calculating *R*-factors(gt) *etc*. and is not relevant to the choice of reflections for refinement. *R*-factors based on *F*^2^ are statistically about twice as large as those based on *F*, and *R*- factors based on ALL data will be even larger.

### Fractional atomic coordinates and isotropic or equivalent isotropic displacement parameters (Å^2^)

**Table:**

	*x*	*y*	*z*	*U*_iso_*/*U*_eq_	
O1A	0.7011 (2)	1.28351 (14)	0.93924 (11)	0.0334 (5)	
O2A	0.7089 (2)	1.14559 (15)	0.98730 (11)	0.0325 (5)	
H2O	0.700 (3)	1.190 (2)	1.0269 (14)	0.049*	
C1A	0.6787 (3)	0.9784 (2)	0.45311 (15)	0.0231 (6)	
C2A	0.6764 (3)	0.8741 (2)	0.45537 (15)	0.0270 (6)	
H2A	0.6868	0.8584	0.5025	0.032*	
C3A	0.6597 (3)	0.7931 (2)	0.39162 (15)	0.0273 (6)	
H3A	0.6569	0.7231	0.3956	0.033*	
C4A	0.6467 (2)	0.8129 (2)	0.32122 (15)	0.0217 (6)	
C5A	0.6330 (3)	0.7317 (2)	0.25410 (15)	0.0262 (6)	
H5A	0.6293	0.6610	0.2567	0.031*	
C6A	0.6252 (3)	0.7529 (2)	0.18704 (16)	0.0285 (6)	
H6A	0.6174	0.6973	0.1437	0.034*	
C7A	0.6286 (2)	0.8582 (2)	0.18038 (15)	0.0227 (6)	
C8A	0.6204 (3)	0.8833 (2)	0.11204 (16)	0.0307 (7)	
H8A	0.6140	0.8292	0.0681	0.037*	
C9A	0.6215 (3)	0.9859 (2)	0.10714 (16)	0.0327 (7)	
H9A	0.6178	1.0014	0.0603	0.039*	
C10A	0.6281 (3)	1.0657 (2)	0.17000 (16)	0.0290 (7)	
H10A	0.6262	1.1352	0.1658	0.035*	
C11A	0.6375 (2)	1.0444 (2)	0.23970 (15)	0.0228 (6)	
C12A	0.6431 (3)	1.1248 (2)	0.30620 (15)	0.0259 (6)	
H12A	0.6378	1.1940	0.3025	0.031*	
C13A	0.6556 (3)	1.1044 (2)	0.37389 (15)	0.0235 (6)	
H13A	0.6598	1.1598	0.4166	0.028*	
C14A	0.6628 (2)	1.0011 (2)	0.38260 (14)	0.0199 (6)	
C15A	0.6496 (2)	0.91896 (19)	0.31704 (14)	0.0190 (6)	
C16A	0.6386 (2)	0.9403 (2)	0.24570 (14)	0.0203 (6)	
C17A	0.6997 (3)	1.0615 (2)	0.52346 (16)	0.0299 (7)	
H17A	0.7488	1.1286	0.5272	0.036*	
C18A	0.6569 (3)	1.0513 (2)	0.58107 (15)	0.0292 (7)	
H18A	0.6067	0.9841	0.5762	0.035*	
C19A	0.6775 (3)	1.1329 (2)	0.65390 (15)	0.0256 (6)	
H19A	0.6015	1.1670	0.6512	0.031*	
H19B	0.7553	1.1878	0.6613	0.031*	
C20A	0.6958 (3)	1.08693 (19)	0.72159 (14)	0.0214 (6)	
H20A	0.6226	1.0268	0.7117	0.026*	
H20B	0.7772	1.0597	0.7276	0.026*	
C21A	0.7024 (3)	1.1674 (2)	0.79478 (14)	0.0219 (6)	
H21A	0.6236	1.1980	0.7880	0.026*	
H21B	0.7792	1.2252	0.8068	0.026*	
C22A	0.7118 (3)	1.1178 (2)	0.86038 (14)	0.0237 (6)	
H22A	0.6393	1.0559	0.8460	0.028*	
H22B	0.7943	1.0923	0.8693	0.028*	
C23A	0.7074 (3)	1.1910 (2)	0.93228 (15)	0.0248 (6)	
O1B	1.0145 (2)	1.00042 (15)	0.91661 (11)	0.0400 (5)	
O2B	0.9862 (2)	0.85927 (15)	0.95987 (11)	0.0336 (5)	
H2P	0.980 (3)	0.906 (2)	0.9987 (14)	0.050*	
C1B	1.0025 (2)	0.7018 (2)	0.43238 (15)	0.0218 (6)	
C2B	1.0249 (2)	0.6030 (2)	0.43784 (15)	0.0237 (6)	
H2B	1.0365	0.5880	0.4853	0.028*	
C3B	1.0308 (2)	0.5264 (2)	0.37656 (15)	0.0236 (6)	
H3B	1.0465	0.4602	0.3826	0.028*	
C4B	1.0140 (2)	0.5448 (2)	0.30571 (15)	0.0215 (6)	
C5B	1.0173 (3)	0.4662 (2)	0.24065 (15)	0.0256 (6)	
H5B	1.0297	0.3987	0.2454	0.031*	
C6B	1.0032 (3)	0.4859 (2)	0.17310 (16)	0.0274 (6)	
H6B	1.0057	0.4320	0.1311	0.033*	
C7B	0.9844 (2)	0.5867 (2)	0.16315 (15)	0.0235 (6)	
C8B	0.9707 (3)	0.6100 (2)	0.09403 (16)	0.0311 (7)	
H8B	0.9738	0.5575	0.0514	0.037*	
C9B	0.9528 (3)	0.7079 (2)	0.08623 (16)	0.0310 (7)	
H9B	0.9438	0.7219	0.0386	0.037*	
C10B	0.9478 (3)	0.7860 (2)	0.14783 (15)	0.0269 (6)	
H10B	0.9352	0.8530	0.1419	0.032*	
C11B	0.9612 (2)	0.7668 (2)	0.21823 (15)	0.0226 (6)	
C12B	0.9575 (3)	0.8450 (2)	0.28320 (15)	0.0245 (6)	
H12B	0.9458	0.9127	0.2785	0.029*	
C13B	0.9702 (2)	0.8255 (2)	0.35100 (15)	0.0234 (6)	
H13B	0.9671	0.8797	0.3926	0.028*	
C14B	0.9885 (2)	0.7246 (2)	0.36191 (15)	0.0203 (6)	
C15B	0.9934 (2)	0.6457 (2)	0.29844 (14)	0.0193 (6)	
C16B	0.9798 (2)	0.6661 (2)	0.22665 (14)	0.0198 (6)	
C17B	0.9926 (3)	0.7791 (2)	0.49945 (16)	0.0309 (7)	
H17B	0.9586	0.8381	0.4901	0.037*	
C18B	1.0226 (4)	0.7777 (3)	0.56672 (18)	0.0535 (10)	
H18B	1.0583	0.7193	0.5758	0.064*	
C19B	1.0111 (3)	0.8540 (2)	0.63549 (16)	0.0334 (7)	
H19C	0.9291	0.8792	0.6229	0.040*	
H19D	1.0843	0.9157	0.6513	0.040*	
C20B	1.0123 (3)	0.8049 (2)	0.70078 (15)	0.0267 (6)	
H20C	0.9357	0.7460	0.6859	0.032*	
H20D	1.0914	0.7752	0.7109	0.032*	
C21B	1.0097 (3)	0.8827 (2)	0.77258 (14)	0.0251 (6)	
H21C	0.9310	0.9129	0.7626	0.030*	
H21D	1.0868	0.9412	0.7880	0.030*	
C22B	1.0095 (3)	0.8319 (2)	0.83661 (15)	0.0259 (6)	
H22C	0.9338	0.7721	0.8204	0.031*	
H22D	1.0894	0.8032	0.8471	0.031*	
C23B	1.0035 (3)	0.9059 (2)	0.90779 (15)	0.0260 (6)	
O1C	0.6907 (2)	0.28109 (14)	0.10986 (11)	0.0331 (5)	
O2C	0.6698 (2)	0.41463 (15)	0.05805 (11)	0.0333 (5)	
H2Q	0.686 (3)	0.371 (2)	0.0220 (15)	0.050*	
C1C	0.6603 (2)	0.5787 (2)	0.58619 (15)	0.0215 (6)	
C2C	0.6594 (3)	0.6831 (2)	0.58354 (15)	0.0244 (6)	
H2C	0.6584	0.7003	0.5375	0.029*	
C3C	0.6598 (3)	0.7614 (2)	0.64555 (15)	0.0253 (6)	
H3C	0.6584	0.8309	0.6414	0.030*	
C4C	0.6622 (2)	0.7396 (2)	0.71438 (15)	0.0221 (6)	
C5C	0.6637 (3)	0.8190 (2)	0.78027 (15)	0.0249 (6)	
H5C	0.6615	0.8887	0.7770	0.030*	
C6C	0.6683 (3)	0.7973 (2)	0.84681 (15)	0.0252 (6)	
H6C	0.6701	0.8520	0.8894	0.030*	
C7C	0.6705 (3)	0.6923 (2)	0.85436 (15)	0.0241 (6)	
C8C	0.6765 (3)	0.6669 (2)	0.92237 (15)	0.0283 (6)	
H8C	0.6799	0.7206	0.9659	0.034*	
C9C	0.6775 (3)	0.5650 (2)	0.92765 (16)	0.0300 (7)	
H9C	0.6822	0.5497	0.9746	0.036*	
C10C	0.6719 (3)	0.4855 (2)	0.86485 (15)	0.0276 (6)	
H10C	0.6722	0.4158	0.8689	0.033*	
C11C	0.6658 (2)	0.5068 (2)	0.79512 (15)	0.0224 (6)	
C12C	0.6606 (3)	0.4266 (2)	0.72900 (15)	0.0232 (6)	
H12C	0.6585	0.3562	0.7319	0.028*	
C13C	0.6589 (2)	0.4486 (2)	0.66270 (15)	0.0226 (6)	
H13C	0.6563	0.3933	0.6203	0.027*	
C14C	0.6607 (2)	0.5538 (2)	0.65459 (14)	0.0194 (6)	
C15C	0.6627 (2)	0.63459 (19)	0.71920 (14)	0.0185 (5)	
C16C	0.6667 (2)	0.6115 (2)	0.78970 (14)	0.0205 (6)	
C17C	0.6560 (3)	0.4983 (2)	0.51713 (15)	0.0234 (6)	
H17C	0.6112	0.4286	0.5108	0.028*	
C18C	0.7091 (3)	0.5148 (2)	0.46341 (15)	0.0239 (6)	
H18C	0.7573	0.5836	0.4707	0.029*	
C19C	0.6997 (3)	0.4343 (2)	0.39215 (14)	0.0237 (6)	
H19E	0.6261	0.3750	0.3836	0.028*	
H19F	0.7805	0.4062	0.3977	0.028*	
C20C	0.6798 (2)	0.47802 (19)	0.32339 (14)	0.0210 (6)	
H20E	0.5960	0.5017	0.3158	0.025*	
H20F	0.7502	0.5403	0.3333	0.025*	
C21C	0.6791 (3)	0.3979 (2)	0.25162 (14)	0.0214 (6)	
H21E	0.7603	0.3708	0.2600	0.026*	
H21F	0.6050	0.3377	0.2396	0.026*	
C22C	0.6679 (3)	0.4450 (2)	0.18521 (14)	0.0223 (6)	
H22E	0.5835	0.4673	0.1748	0.027*	
H22F	0.7379	0.5088	0.1993	0.027*	
C23C	0.6770 (3)	0.3718 (2)	0.11480 (15)	0.0237 (6)	

### Atomic displacement parameters (Å^2^)

**Table:**

	*U*^11^	*U*^22^	*U*^33^	*U*^12^	*U*^13^	*U*^23^
O1A	0.0587 (14)	0.0231 (11)	0.0227 (11)	0.0115 (9)	0.0163 (10)	0.0075 (9)
O2A	0.0563 (14)	0.0268 (11)	0.0182 (11)	0.0132 (10)	0.0130 (10)	0.0076 (9)
C1A	0.0248 (14)	0.0241 (14)	0.0190 (14)	0.0040 (11)	0.0060 (11)	0.0038 (12)
C2A	0.0362 (16)	0.0286 (15)	0.0199 (15)	0.0088 (12)	0.0081 (12)	0.0120 (13)
C3A	0.0353 (16)	0.0219 (14)	0.0268 (16)	0.0095 (12)	0.0078 (13)	0.0091 (13)
C4A	0.0212 (14)	0.0228 (14)	0.0225 (15)	0.0073 (11)	0.0063 (11)	0.0066 (12)
C5A	0.0285 (15)	0.0217 (14)	0.0281 (16)	0.0078 (11)	0.0081 (12)	0.0045 (12)
C6A	0.0286 (15)	0.0276 (15)	0.0268 (16)	0.0064 (12)	0.0084 (12)	0.0014 (13)
C7A	0.0197 (13)	0.0297 (15)	0.0185 (14)	0.0050 (11)	0.0065 (11)	0.0048 (12)
C8A	0.0337 (16)	0.0374 (17)	0.0203 (15)	0.0076 (13)	0.0103 (12)	0.0032 (13)
C9A	0.0357 (17)	0.0419 (18)	0.0247 (16)	0.0059 (13)	0.0103 (13)	0.0163 (14)
C10A	0.0328 (16)	0.0297 (15)	0.0262 (16)	0.0032 (12)	0.0102 (13)	0.0107 (13)
C11A	0.0241 (14)	0.0246 (14)	0.0193 (14)	0.0027 (11)	0.0067 (11)	0.0062 (12)
C12A	0.0290 (15)	0.0220 (14)	0.0282 (16)	0.0050 (11)	0.0078 (12)	0.0102 (13)
C13A	0.0277 (15)	0.0216 (14)	0.0213 (15)	0.0061 (11)	0.0079 (12)	0.0043 (12)
C14A	0.0192 (13)	0.0222 (13)	0.0184 (14)	0.0053 (10)	0.0055 (11)	0.0047 (11)
C15A	0.0156 (13)	0.0214 (13)	0.0203 (14)	0.0043 (10)	0.0058 (10)	0.0052 (11)
C16A	0.0169 (13)	0.0256 (14)	0.0192 (14)	0.0058 (10)	0.0064 (11)	0.0055 (12)
C17A	0.0389 (17)	0.0257 (15)	0.0235 (16)	0.0039 (12)	0.0071 (13)	0.0066 (13)
C18A	0.0396 (17)	0.0254 (15)	0.0233 (16)	0.0059 (12)	0.0091 (13)	0.0080 (13)
C19A	0.0310 (15)	0.0249 (14)	0.0211 (15)	0.0063 (12)	0.0093 (12)	0.0043 (12)
C20A	0.0252 (14)	0.0190 (13)	0.0202 (14)	0.0050 (10)	0.0072 (11)	0.0040 (11)
C21A	0.0250 (14)	0.0225 (13)	0.0192 (14)	0.0062 (11)	0.0072 (11)	0.0057 (12)
C22A	0.0305 (15)	0.0216 (13)	0.0196 (14)	0.0068 (11)	0.0072 (12)	0.0059 (12)
C23A	0.0270 (15)	0.0269 (15)	0.0217 (15)	0.0050 (11)	0.0067 (12)	0.0089 (12)
O1B	0.0757 (16)	0.0215 (11)	0.0268 (12)	0.0097 (10)	0.0225 (11)	0.0060 (9)
O2B	0.0565 (14)	0.0276 (11)	0.0198 (11)	0.0080 (10)	0.0149 (10)	0.0086 (9)
C1B	0.0195 (13)	0.0273 (14)	0.0180 (14)	0.0038 (11)	0.0049 (11)	0.0056 (12)
C2B	0.0238 (14)	0.0286 (15)	0.0204 (15)	0.0080 (11)	0.0053 (11)	0.0091 (12)
C3B	0.0214 (14)	0.0228 (14)	0.0275 (16)	0.0052 (11)	0.0058 (12)	0.0088 (12)
C4B	0.0162 (13)	0.0240 (14)	0.0229 (15)	0.0026 (10)	0.0048 (11)	0.0049 (12)
C5B	0.0274 (15)	0.0218 (14)	0.0274 (16)	0.0067 (11)	0.0087 (12)	0.0039 (12)
C6B	0.0287 (15)	0.0261 (15)	0.0235 (16)	0.0047 (12)	0.0091 (12)	−0.0021 (12)
C7B	0.0200 (14)	0.0271 (15)	0.0206 (15)	0.0010 (11)	0.0068 (11)	0.0024 (12)
C8B	0.0336 (16)	0.0350 (16)	0.0206 (15)	0.0020 (13)	0.0081 (13)	0.0021 (13)
C9B	0.0345 (16)	0.0401 (17)	0.0199 (15)	0.0040 (13)	0.0072 (12)	0.0134 (14)
C10B	0.0280 (15)	0.0291 (15)	0.0253 (16)	0.0038 (12)	0.0070 (12)	0.0126 (13)
C11B	0.0212 (14)	0.0235 (14)	0.0234 (15)	0.0022 (11)	0.0056 (11)	0.0087 (12)
C12B	0.0276 (15)	0.0212 (14)	0.0267 (16)	0.0064 (11)	0.0081 (12)	0.0089 (12)
C13B	0.0235 (14)	0.0233 (14)	0.0230 (15)	0.0059 (11)	0.0069 (11)	0.0042 (12)
C14B	0.0168 (13)	0.0228 (14)	0.0194 (14)	0.0038 (10)	0.0030 (11)	0.0041 (12)
C15B	0.0141 (12)	0.0227 (14)	0.0202 (14)	0.0025 (10)	0.0050 (10)	0.0048 (12)
C16B	0.0175 (13)	0.0242 (14)	0.0165 (14)	0.0014 (10)	0.0054 (10)	0.0040 (11)
C17B	0.0482 (19)	0.0283 (15)	0.0240 (17)	0.0186 (13)	0.0136 (14)	0.0117 (13)
C18B	0.117 (3)	0.0291 (17)	0.0253 (19)	0.0310 (19)	0.0275 (19)	0.0113 (15)
C19B	0.054 (2)	0.0273 (15)	0.0196 (15)	0.0114 (14)	0.0116 (14)	0.0049 (13)
C20B	0.0342 (16)	0.0261 (14)	0.0199 (15)	0.0077 (12)	0.0054 (12)	0.0076 (12)
C21B	0.0305 (15)	0.0245 (14)	0.0193 (15)	0.0050 (12)	0.0069 (12)	0.0042 (12)
C22B	0.0310 (15)	0.0261 (15)	0.0221 (15)	0.0084 (12)	0.0074 (12)	0.0075 (12)
C23B	0.0307 (15)	0.0267 (15)	0.0207 (15)	0.0039 (12)	0.0072 (12)	0.0074 (12)
O1C	0.0585 (14)	0.0228 (11)	0.0225 (11)	0.0117 (9)	0.0162 (10)	0.0077 (9)
O2C	0.0568 (14)	0.0284 (11)	0.0212 (11)	0.0152 (10)	0.0163 (10)	0.0098 (9)
C1C	0.0195 (13)	0.0235 (14)	0.0207 (15)	0.0039 (11)	0.0054 (11)	0.0049 (12)
C2C	0.0293 (15)	0.0260 (14)	0.0201 (15)	0.0060 (11)	0.0073 (12)	0.0094 (12)
C3C	0.0311 (15)	0.0202 (14)	0.0262 (16)	0.0068 (11)	0.0092 (12)	0.0070 (12)
C4C	0.0242 (14)	0.0202 (13)	0.0231 (15)	0.0062 (11)	0.0068 (11)	0.0069 (12)
C5C	0.0278 (15)	0.0199 (14)	0.0269 (16)	0.0073 (11)	0.0082 (12)	0.0040 (12)
C6C	0.0275 (15)	0.0231 (14)	0.0235 (15)	0.0067 (11)	0.0085 (12)	0.0008 (12)
C7C	0.0240 (14)	0.0264 (14)	0.0215 (15)	0.0048 (11)	0.0069 (11)	0.0054 (12)
C8C	0.0334 (16)	0.0340 (16)	0.0160 (14)	0.0065 (12)	0.0078 (12)	0.0032 (12)
C9C	0.0331 (16)	0.0394 (17)	0.0178 (15)	0.0062 (13)	0.0050 (12)	0.0114 (13)
C10C	0.0330 (16)	0.0300 (15)	0.0236 (16)	0.0083 (12)	0.0095 (12)	0.0120 (13)
C11C	0.0212 (14)	0.0252 (14)	0.0228 (15)	0.0054 (11)	0.0070 (11)	0.0093 (12)
C12C	0.0274 (15)	0.0203 (13)	0.0234 (15)	0.0062 (11)	0.0085 (12)	0.0067 (12)
C13C	0.0268 (14)	0.0183 (13)	0.0217 (15)	0.0052 (11)	0.0088 (12)	0.0009 (11)
C14C	0.0175 (13)	0.0239 (13)	0.0175 (14)	0.0033 (10)	0.0062 (11)	0.0063 (11)
C15C	0.0168 (13)	0.0206 (13)	0.0171 (14)	0.0026 (10)	0.0043 (10)	0.0043 (11)
C16C	0.0176 (13)	0.0242 (14)	0.0193 (14)	0.0039 (11)	0.0049 (11)	0.0054 (12)
C17C	0.0265 (15)	0.0229 (14)	0.0201 (15)	0.0055 (11)	0.0049 (11)	0.0057 (12)
C18C	0.0277 (15)	0.0216 (13)	0.0208 (15)	0.0044 (11)	0.0056 (12)	0.0040 (11)
C19C	0.0298 (15)	0.0239 (14)	0.0194 (14)	0.0081 (11)	0.0085 (12)	0.0062 (12)
C20C	0.0234 (14)	0.0218 (13)	0.0188 (14)	0.0054 (11)	0.0070 (11)	0.0057 (11)
C21C	0.0230 (14)	0.0229 (13)	0.0179 (14)	0.0059 (11)	0.0048 (11)	0.0050 (11)
C22C	0.0264 (14)	0.0231 (14)	0.0177 (14)	0.0050 (11)	0.0071 (11)	0.0050 (12)
C23C	0.0264 (14)	0.0255 (15)	0.0183 (14)	0.0030 (11)	0.0054 (11)	0.0063 (12)

### Geometric parameters (Å, °)

**Table:**

O1A—C23A	1.224 (3)	C11B—C16B	1.425 (4)
O2A—C23A	1.319 (3)	C11B—C12B	1.432 (4)
O2A—H2O	0.875 (18)	C12B—C13B	1.350 (4)
C1A—C2A	1.398 (4)	C12B—H12B	0.9500
C1A—C14A	1.416 (4)	C13B—C14B	1.441 (3)
C1A—C17A	1.472 (4)	C13B—H13B	0.9500
C2A—C3A	1.380 (4)	C14B—C15B	1.422 (4)
C2A—H2A	0.9500	C15B—C16B	1.428 (3)
C3A—C4A	1.399 (4)	C17B—C18B	1.248 (4)
C3A—H3A	0.9500	C17B—H17B	0.9500
C4A—C5A	1.431 (4)	C18B—C19B	1.500 (4)
C4A—C15A	1.431 (3)	C18B—H18B	0.9500
C5A—C6A	1.352 (4)	C19B—C20B	1.526 (4)
C5A—H5A	0.9500	C19B—H19C	0.9900
C6A—C7A	1.434 (4)	C19B—H19D	0.9900
C6A—H6A	0.9500	C20B—C21B	1.519 (4)
C7A—C8A	1.398 (4)	C20B—H20C	0.9900
C7A—C16A	1.423 (4)	C20B—H20D	0.9900
C8A—C9A	1.391 (4)	C21B—C22B	1.520 (3)
C8A—H8A	0.9500	C21B—H21C	0.9900
C9A—C10A	1.383 (4)	C21B—H21D	0.9900
C9A—H9A	0.9500	C22B—C23B	1.498 (4)
C10A—C11A	1.399 (4)	C22B—H22C	0.9900
C10A—H10A	0.9500	C22B—H22D	0.9900
C11A—C16A	1.420 (3)	O1C—C23C	1.224 (3)
C11A—C12A	1.437 (4)	O2C—C23C	1.322 (3)
C12A—C13A	1.353 (4)	O2C—H2Q	0.859 (18)
C12A—H12A	0.9500	C1C—C2C	1.405 (4)
C13A—C14A	1.435 (3)	C1C—C14C	1.410 (3)
C13A—H13A	0.9500	C1C—C17C	1.476 (4)
C14A—C15A	1.419 (4)	C2C—C3C	1.380 (4)
C15A—C16A	1.428 (4)	C2C—H2C	0.9500
C17A—C18A	1.304 (4)	C3C—C4C	1.396 (4)
C17A—H17A	0.9500	C3C—H3C	0.9500
C18A—C19A	1.497 (4)	C4C—C15C	1.424 (3)
C18A—H18A	0.9500	C4C—C5C	1.435 (4)
C19A—C20A	1.531 (3)	C5C—C6C	1.350 (4)
C19A—H19A	0.9900	C5C—H5C	0.9500
C19A—H19B	0.9900	C6C—C7C	1.442 (4)
C20A—C21A	1.526 (4)	C6C—H6C	0.9500
C20A—H20A	0.9900	C7C—C8C	1.397 (4)
C20A—H20B	0.9900	C7C—C16C	1.423 (4)
C21A—C22A	1.527 (3)	C8C—C9C	1.387 (4)
C21A—H21A	0.9900	C8C—H8C	0.9500
C21A—H21B	0.9900	C9C—C10C	1.383 (4)
C22A—C23A	1.498 (4)	C9C—H9C	0.9500
C22A—H22A	0.9900	C10C—C11C	1.404 (4)
C22A—H22B	0.9900	C10C—H10C	0.9500
O1B—C23B	1.217 (3)	C11C—C16C	1.421 (3)
O2B—C23B	1.314 (3)	C11C—C12C	1.433 (4)
O2B—H2P	0.872 (18)	C12C—C13C	1.351 (4)
C1B—C2B	1.396 (4)	C12C—H12C	0.9500
C1B—C14B	1.417 (4)	C13C—C14C	1.444 (3)
C1B—C17B	1.477 (4)	C13C—H13C	0.9500
C2B—C3B	1.379 (4)	C14C—C15C	1.427 (4)
C2B—H2B	0.9500	C15C—C16C	1.432 (3)
C3B—C4B	1.397 (4)	C17C—C18C	1.320 (4)
C3B—H3B	0.9500	C17C—H17C	0.9500
C4B—C15B	1.426 (4)	C18C—C19C	1.492 (4)
C4B—C5B	1.434 (4)	C18C—H18C	0.9500
C5B—C6B	1.345 (4)	C19C—C20C	1.531 (3)
C5B—H5B	0.9500	C19C—H19E	0.9900
C6B—C7B	1.435 (4)	C19C—H19F	0.9900
C6B—H6B	0.9500	C20C—C21C	1.522 (4)
C7B—C8B	1.396 (4)	C20C—H20E	0.9900
C7B—C16B	1.424 (4)	C20C—H20F	0.9900
C8B—C9B	1.383 (4)	C21C—C22C	1.520 (3)
C8B—H8B	0.9500	C21C—H21E	0.9900
C9B—C10B	1.391 (4)	C21C—H21F	0.9900
C9B—H9B	0.9500	C22C—C23C	1.494 (4)
C10B—C11B	1.396 (4)	C22C—H22E	0.9900
C10B—H10B	0.9500	C22C—H22F	0.9900
			
C23A—O2A—H2O	110 (2)	C1B—C14B—C13B	123.1 (2)
C2A—C1A—C14A	118.5 (2)	C15B—C14B—C13B	117.5 (2)
C2A—C1A—C17A	119.6 (2)	C14B—C15B—C4B	120.4 (2)
C14A—C1A—C17A	121.9 (2)	C14B—C15B—C16B	120.8 (2)
C3A—C2A—C1A	122.4 (2)	C4B—C15B—C16B	118.8 (2)
C3A—C2A—H2A	118.8	C7B—C16B—C11B	119.5 (2)
C1A—C2A—H2A	118.8	C7B—C16B—C15B	120.6 (2)
C2A—C3A—C4A	120.8 (2)	C11B—C16B—C15B	119.8 (2)
C2A—C3A—H3A	119.6	C18B—C17B—C1B	129.0 (3)
C4A—C3A—H3A	119.6	C18B—C17B—H17B	115.5
C3A—C4A—C5A	122.6 (2)	C1B—C17B—H17B	115.5
C3A—C4A—C15A	118.2 (2)	C17B—C18B—C19B	130.0 (3)
C5A—C4A—C15A	119.2 (2)	C17B—C18B—H18B	115.0
C6A—C5A—C4A	121.7 (2)	C19B—C18B—H18B	115.0
C6A—C5A—H5A	119.1	C18B—C19B—C20B	112.4 (2)
C4A—C5A—H5A	119.1	C18B—C19B—H19C	109.1
C5A—C6A—C7A	120.9 (3)	C20B—C19B—H19C	109.1
C5A—C6A—H6A	119.5	C18B—C19B—H19D	109.1
C7A—C6A—H6A	119.5	C20B—C19B—H19D	109.1
C8A—C7A—C16A	118.6 (2)	H19C—C19B—H19D	107.9
C8A—C7A—C6A	122.7 (3)	C21B—C20B—C19B	113.2 (2)
C16A—C7A—C6A	118.7 (2)	C21B—C20B—H20C	108.9
C9A—C8A—C7A	121.3 (3)	C19B—C20B—H20C	108.9
C9A—C8A—H8A	119.3	C21B—C20B—H20D	108.9
C7A—C8A—H8A	119.3	C19B—C20B—H20D	108.9
C10A—C9A—C8A	120.5 (3)	H20C—C20B—H20D	107.7
C10A—C9A—H9A	119.8	C20B—C21B—C22B	112.3 (2)
C8A—C9A—H9A	119.8	C20B—C21B—H21C	109.1
C9A—C10A—C11A	120.2 (3)	C22B—C21B—H21C	109.1
C9A—C10A—H10A	119.9	C20B—C21B—H21D	109.1
C11A—C10A—H10A	119.9	C22B—C21B—H21D	109.1
C10A—C11A—C16A	119.9 (2)	H21C—C21B—H21D	107.9
C10A—C11A—C12A	122.0 (2)	C23B—C22B—C21B	113.8 (2)
C16A—C11A—C12A	118.1 (2)	C23B—C22B—H22C	108.8
C13A—C12A—C11A	121.8 (2)	C21B—C22B—H22C	108.8
C13A—C12A—H12A	119.1	C23B—C22B—H22D	108.8
C11A—C12A—H12A	119.1	C21B—C22B—H22D	108.8
C12A—C13A—C14A	121.5 (2)	H22C—C22B—H22D	107.7
C12A—C13A—H13A	119.2	O1B—C23B—O2B	123.2 (3)
C14A—C13A—H13A	119.2	O1B—C23B—C22B	123.0 (2)
C1A—C14A—C15A	119.6 (2)	O2B—C23B—C22B	113.8 (2)
C1A—C14A—C13A	122.6 (2)	C23C—O2C—H2Q	107 (2)
C15A—C14A—C13A	117.8 (2)	C2C—C1C—C14C	118.4 (2)
C14A—C15A—C16A	120.8 (2)	C2C—C1C—C17C	119.3 (2)
C14A—C15A—C4A	120.5 (2)	C14C—C1C—C17C	122.2 (2)
C16A—C15A—C4A	118.7 (2)	C3C—C2C—C1C	122.2 (2)
C11A—C16A—C7A	119.6 (2)	C3C—C2C—H2C	118.9
C11A—C16A—C15A	119.8 (2)	C1C—C2C—H2C	118.9
C7A—C16A—C15A	120.7 (2)	C2C—C3C—C4C	120.7 (2)
C18A—C17A—C1A	126.0 (3)	C2C—C3C—H3C	119.7
C18A—C17A—H17A	117.0	C4C—C3C—H3C	119.7
C1A—C17A—H17A	117.0	C3C—C4C—C15C	118.7 (2)
C17A—C18A—C19A	127.7 (3)	C3C—C4C—C5C	122.4 (2)
C17A—C18A—H18A	116.1	C15C—C4C—C5C	118.9 (2)
C19A—C18A—H18A	116.1	C6C—C5C—C4C	121.8 (2)
C18A—C19A—C20A	112.7 (2)	C6C—C5C—H5C	119.1
C18A—C19A—H19A	109.0	C4C—C5C—H5C	119.1
C20A—C19A—H19A	109.0	C5C—C6C—C7C	121.1 (2)
C18A—C19A—H19B	109.0	C5C—C6C—H6C	119.5
C20A—C19A—H19B	109.0	C7C—C6C—H6C	119.5
H19A—C19A—H19B	107.8	C8C—C7C—C16C	118.7 (2)
C21A—C20A—C19A	112.9 (2)	C8C—C7C—C6C	122.8 (2)
C21A—C20A—H20A	109.0	C16C—C7C—C6C	118.4 (2)
C19A—C20A—H20A	109.0	C9C—C8C—C7C	121.4 (3)
C21A—C20A—H20B	109.0	C9C—C8C—H8C	119.3
C19A—C20A—H20B	109.0	C7C—C8C—H8C	119.3
H20A—C20A—H20B	107.8	C10C—C9C—C8C	120.3 (3)
C20A—C21A—C22A	111.9 (2)	C10C—C9C—H9C	119.9
C20A—C21A—H21A	109.2	C8C—C9C—H9C	119.9
C22A—C21A—H21A	109.2	C9C—C10C—C11C	120.6 (3)
C20A—C21A—H21B	109.2	C9C—C10C—H10C	119.7
C22A—C21A—H21B	109.2	C11C—C10C—H10C	119.7
H21A—C21A—H21B	107.9	C10C—C11C—C16C	119.3 (2)
C23A—C22A—C21A	114.2 (2)	C10C—C11C—C12C	122.1 (2)
C23A—C22A—H22A	108.7	C16C—C11C—C12C	118.5 (2)
C21A—C22A—H22A	108.7	C13C—C12C—C11C	121.7 (2)
C23A—C22A—H22B	108.7	C13C—C12C—H12C	119.2
C21A—C22A—H22B	108.7	C11C—C12C—H12C	119.2
H22A—C22A—H22B	107.6	C12C—C13C—C14C	121.8 (2)
O1A—C23A—O2A	123.1 (3)	C12C—C13C—H13C	119.1
O1A—C23A—C22A	123.5 (2)	C14C—C13C—H13C	119.1
O2A—C23A—C22A	113.5 (2)	C1C—C14C—C15C	119.7 (2)
C23B—O2B—H2P	109 (2)	C1C—C14C—C13C	122.7 (2)
C2B—C1B—C14B	118.7 (2)	C15C—C14C—C13C	117.6 (2)
C2B—C1B—C17B	119.5 (2)	C4C—C15C—C14C	120.2 (2)
C14B—C1B—C17B	121.8 (2)	C4C—C15C—C16C	119.3 (2)
C3B—C2B—C1B	122.1 (2)	C14C—C15C—C16C	120.5 (2)
C3B—C2B—H2B	119.0	C11C—C16C—C7C	119.6 (2)
C1B—C2B—H2B	119.0	C11C—C16C—C15C	119.9 (2)
C2B—C3B—C4B	121.0 (2)	C7C—C16C—C15C	120.5 (2)
C2B—C3B—H3B	119.5	C18C—C17C—C1C	125.7 (2)
C4B—C3B—H3B	119.5	C18C—C17C—H17C	117.2
C3B—C4B—C15B	118.4 (2)	C1C—C17C—H17C	117.2
C3B—C4B—C5B	122.3 (2)	C17C—C18C—C19C	125.5 (2)
C15B—C4B—C5B	119.3 (2)	C17C—C18C—H18C	117.2
C6B—C5B—C4B	121.5 (2)	C19C—C18C—H18C	117.2
C6B—C5B—H5B	119.2	C18C—C19C—C20C	113.1 (2)
C4B—C5B—H5B	119.2	C18C—C19C—H19E	109.0
C5B—C6B—C7B	121.3 (3)	C20C—C19C—H19E	109.0
C5B—C6B—H6B	119.3	C18C—C19C—H19F	109.0
C7B—C6B—H6B	119.3	C20C—C19C—H19F	109.0
C8B—C7B—C16B	118.8 (2)	H19E—C19C—H19F	107.8
C8B—C7B—C6B	122.8 (3)	C21C—C20C—C19C	113.1 (2)
C16B—C7B—C6B	118.4 (2)	C21C—C20C—H20E	109.0
C9B—C8B—C7B	121.4 (3)	C19C—C20C—H20E	109.0
C9B—C8B—H8B	119.3	C21C—C20C—H20F	109.0
C7B—C8B—H8B	119.3	C19C—C20C—H20F	109.0
C8B—C9B—C10B	120.3 (3)	H20E—C20C—H20F	107.8
C8B—C9B—H9B	119.9	C22C—C21C—C20C	112.2 (2)
C10B—C9B—H9B	119.9	C22C—C21C—H21E	109.2
C9B—C10B—C11B	120.6 (3)	C20C—C21C—H21E	109.2
C9B—C10B—H10B	119.7	C22C—C21C—H21F	109.2
C11B—C10B—H10B	119.7	C20C—C21C—H21F	109.2
C10B—C11B—C16B	119.4 (2)	H21E—C21C—H21F	107.9
C10B—C11B—C12B	122.4 (2)	C23C—C22C—C21C	114.2 (2)
C16B—C11B—C12B	118.2 (2)	C23C—C22C—H22E	108.7
C13B—C12B—C11B	121.9 (2)	C21C—C22C—H22E	108.7
C13B—C12B—H12B	119.0	C23C—C22C—H22F	108.7
C11B—C12B—H12B	119.0	C21C—C22C—H22F	108.7
C12B—C13B—C14B	121.7 (2)	H22E—C22C—H22F	107.6
C12B—C13B—H13B	119.1	O1C—C23C—O2C	122.9 (2)
C14B—C13B—H13B	119.1	O1C—C23C—C22C	123.6 (2)
C1B—C14B—C15B	119.4 (2)	O2C—C23C—C22C	113.5 (2)
			
C14A—C1A—C2A—C3A	0.2 (4)	C1B—C14B—C15B—C16B	−179.9 (2)
C17A—C1A—C2A—C3A	−178.7 (3)	C13B—C14B—C15B—C16B	−0.4 (3)
C1A—C2A—C3A—C4A	1.3 (4)	C3B—C4B—C15B—C14B	−0.8 (4)
C2A—C3A—C4A—C5A	178.1 (3)	C5B—C4B—C15B—C14B	179.4 (2)
C2A—C3A—C4A—C15A	−0.8 (4)	C3B—C4B—C15B—C16B	178.3 (2)
C3A—C4A—C5A—C6A	−177.6 (3)	C5B—C4B—C15B—C16B	−1.5 (3)
C15A—C4A—C5A—C6A	1.2 (4)	C8B—C7B—C16B—C11B	0.2 (4)
C4A—C5A—C6A—C7A	−0.9 (4)	C6B—C7B—C16B—C11B	−180.0 (2)
C5A—C6A—C7A—C8A	−179.8 (3)	C8B—C7B—C16B—C15B	−180.0 (2)
C5A—C6A—C7A—C16A	−0.8 (4)	C6B—C7B—C16B—C15B	−0.1 (4)
C16A—C7A—C8A—C9A	−0.2 (4)	C10B—C11B—C16B—C7B	0.0 (4)
C6A—C7A—C8A—C9A	178.8 (3)	C12B—C11B—C16B—C7B	−179.8 (2)
C7A—C8A—C9A—C10A	−1.3 (4)	C10B—C11B—C16B—C15B	−179.9 (2)
C8A—C9A—C10A—C11A	1.9 (4)	C12B—C11B—C16B—C15B	0.4 (3)
C9A—C10A—C11A—C16A	−0.8 (4)	C14B—C15B—C16B—C7B	−179.8 (2)
C9A—C10A—C11A—C12A	−179.3 (3)	C4B—C15B—C16B—C7B	1.1 (3)
C10A—C11A—C12A—C13A	−178.2 (3)	C14B—C15B—C16B—C11B	0.1 (3)
C16A—C11A—C12A—C13A	3.3 (4)	C4B—C15B—C16B—C11B	−179.0 (2)
C11A—C12A—C13A—C14A	−0.6 (4)	C2B—C1B—C17B—C18B	13.3 (5)
C2A—C1A—C14A—C15A	−2.1 (4)	C14B—C1B—C17B—C18B	−167.4 (4)
C17A—C1A—C14A—C15A	176.8 (2)	C1B—C17B—C18B—C19B	−178.6 (3)
C2A—C1A—C14A—C13A	176.0 (2)	C17B—C18B—C19B—C20B	159.4 (4)
C17A—C1A—C14A—C13A	−5.1 (4)	C18B—C19B—C20B—C21B	176.2 (3)
C12A—C13A—C14A—C1A	178.7 (3)	C19B—C20B—C21B—C22B	179.4 (2)
C12A—C13A—C14A—C15A	−3.2 (4)	C20B—C21B—C22B—C23B	−178.5 (2)
C1A—C14A—C15A—C16A	−177.5 (2)	C21B—C22B—C23B—O1B	−8.9 (4)
C13A—C14A—C15A—C16A	4.2 (4)	C21B—C22B—C23B—O2B	171.5 (2)
C1A—C14A—C15A—C4A	2.7 (4)	C14C—C1C—C2C—C3C	0.5 (4)
C13A—C14A—C15A—C4A	−175.6 (2)	C17C—C1C—C2C—C3C	178.3 (2)
C3A—C4A—C15A—C14A	−1.2 (4)	C1C—C2C—C3C—C4C	0.5 (4)
C5A—C4A—C15A—C14A	179.9 (2)	C2C—C3C—C4C—C15C	−0.7 (4)
C3A—C4A—C15A—C16A	179.0 (2)	C2C—C3C—C4C—C5C	179.5 (2)
C5A—C4A—C15A—C16A	0.1 (4)	C3C—C4C—C5C—C6C	−178.8 (3)
C10A—C11A—C16A—C7A	−0.8 (4)	C15C—C4C—C5C—C6C	1.4 (4)
C12A—C11A—C16A—C7A	177.8 (2)	C4C—C5C—C6C—C7C	−0.6 (4)
C10A—C11A—C16A—C15A	179.3 (2)	C5C—C6C—C7C—C8C	179.3 (3)
C12A—C11A—C16A—C15A	−2.1 (4)	C5C—C6C—C7C—C16C	−0.5 (4)
C8A—C7A—C16A—C11A	1.3 (4)	C16C—C7C—C8C—C9C	−0.6 (4)
C6A—C7A—C16A—C11A	−177.8 (2)	C6C—C7C—C8C—C9C	179.6 (3)
C8A—C7A—C16A—C15A	−178.8 (2)	C7C—C8C—C9C—C10C	−0.3 (4)
C6A—C7A—C16A—C15A	2.1 (4)	C8C—C9C—C10C—C11C	0.4 (4)
C14A—C15A—C16A—C11A	−1.6 (4)	C9C—C10C—C11C—C16C	0.6 (4)
C4A—C15A—C16A—C11A	178.2 (2)	C9C—C10C—C11C—C12C	179.6 (3)
C14A—C15A—C16A—C7A	178.5 (2)	C10C—C11C—C12C—C13C	−177.9 (3)
C4A—C15A—C16A—C7A	−1.7 (4)	C16C—C11C—C12C—C13C	1.1 (4)
C2A—C1A—C17A—C18A	−35.6 (4)	C11C—C12C—C13C—C14C	−0.6 (4)
C14A—C1A—C17A—C18A	145.6 (3)	C2C—C1C—C14C—C15C	−1.2 (4)
C1A—C17A—C18A—C19A	179.0 (3)	C17C—C1C—C14C—C15C	−178.9 (2)
C17A—C18A—C19A—C20A	−143.0 (3)	C2C—C1C—C14C—C13C	178.7 (2)
C18A—C19A—C20A—C21A	−173.9 (2)	C17C—C1C—C14C—C13C	0.9 (4)
C19A—C20A—C21A—C22A	176.3 (2)	C12C—C13C—C14C—C1C	179.3 (2)
C20A—C21A—C22A—C23A	−175.2 (2)	C12C—C13C—C14C—C15C	−0.8 (4)
C21A—C22A—C23A—O1A	−2.3 (4)	C3C—C4C—C15C—C14C	0.0 (4)
C21A—C22A—C23A—O2A	176.7 (2)	C5C—C4C—C15C—C14C	179.8 (2)
C14B—C1B—C2B—C3B	−1.5 (4)	C3C—C4C—C15C—C16C	179.3 (2)
C17B—C1B—C2B—C3B	177.8 (2)	C5C—C4C—C15C—C16C	−0.9 (4)
C1B—C2B—C3B—C4B	−0.2 (4)	C1C—C14C—C15C—C4C	1.0 (4)
C2B—C3B—C4B—C15B	1.4 (4)	C13C—C14C—C15C—C4C	−178.9 (2)
C2B—C3B—C4B—C5B	−178.9 (2)	C1C—C14C—C15C—C16C	−178.3 (2)
C3B—C4B—C5B—C6B	−178.8 (3)	C13C—C14C—C15C—C16C	1.8 (3)
C15B—C4B—C5B—C6B	0.9 (4)	C10C—C11C—C16C—C7C	−1.6 (4)
C4B—C5B—C6B—C7B	0.1 (4)	C12C—C11C—C16C—C7C	179.4 (2)
C5B—C6B—C7B—C8B	179.3 (3)	C10C—C11C—C16C—C15C	178.9 (2)
C5B—C6B—C7B—C16B	−0.5 (4)	C12C—C11C—C16C—C15C	−0.1 (4)
C16B—C7B—C8B—C9B	−0.1 (4)	C8C—C7C—C16C—C11C	1.6 (4)
C6B—C7B—C8B—C9B	−180.0 (3)	C6C—C7C—C16C—C11C	−178.6 (2)
C7B—C8B—C9B—C10B	−0.1 (4)	C8C—C7C—C16C—C15C	−178.9 (2)
C8B—C9B—C10B—C11B	0.2 (4)	C6C—C7C—C16C—C15C	0.9 (4)
C9B—C10B—C11B—C16B	−0.1 (4)	C4C—C15C—C16C—C11C	179.3 (2)
C9B—C10B—C11B—C12B	179.6 (3)	C14C—C15C—C16C—C11C	−1.4 (4)
C10B—C11B—C12B—C13B	179.8 (3)	C4C—C15C—C16C—C7C	−0.2 (4)
C16B—C11B—C12B—C13B	−0.5 (4)	C14C—C15C—C16C—C7C	179.1 (2)
C11B—C12B—C13B—C14B	0.1 (4)	C2C—C1C—C17C—C18C	33.4 (4)
C2B—C1B—C14B—C15B	2.0 (4)	C14C—C1C—C17C—C18C	−148.9 (3)
C17B—C1B—C14B—C15B	−177.4 (2)	C1C—C17C—C18C—C19C	−177.2 (2)
C2B—C1B—C14B—C13B	−177.5 (2)	C17C—C18C—C19C—C20C	139.5 (3)
C17B—C1B—C14B—C13B	3.2 (4)	C18C—C19C—C20C—C21C	176.3 (2)
C12B—C13B—C14B—C1B	179.8 (2)	C19C—C20C—C21C—C22C	−176.3 (2)
C12B—C13B—C14B—C15B	0.3 (4)	C20C—C21C—C22C—C23C	175.6 (2)
C1B—C14B—C15B—C4B	−0.8 (4)	C21C—C22C—C23C—O1C	0.8 (4)
C13B—C14B—C15B—C4B	178.7 (2)	C21C—C22C—C23C—O2C	−178.7 (2)

### Hydrogen-bond geometry (Å, °)

**Table:**

Cg1 and Cg2 are the centroids of the C1A–C4A,C15A,C14A and C7C–C11C,C16C rings, respectively.

**Table:**

*D*—H···*A*	*D*—H	H···*A*	*D*···*A*	*D*—H···*A*
O2A—H2O···O1C^i^	0.88 (2)	1.79 (2)	2.661 (3)	177 (3)
O2B—H2P···O1B^ii^	0.87 (2)	1.78 (2)	2.642 (3)	173 (3)
O2C—H2Q···O1A^iii^	0.86 (2)	1.78 (2)	2.636 (3)	172 (3)
C10C—H10C···O1A^iv^	0.95	2.46	3.336 (3)	154
C10B—H10B···O1B^v^	0.95	2.51	3.354 (3)	148
C10A—H10A···O1C^vi^	0.95	2.51	3.360 (3)	150
C22C—H22E···Cg2^vii^	0.99	2.63	3.500 (4)	147
C19A—H19A···Cg1^viii^	0.99	2.72	3.511 (4)	137

Symmetry codes: (i) *x*, *y*+1, *z*+1; (ii) −*x*+2, −*y*+2, −*z*+2; (iii) *x*, *y*−1, *z*−1; (iv) *x*, *y*−1, *z*; (v) −*x*+2, −*y*+2, −*z*+1; (vi) *x*, *y*+1, *z*; (vii) −*x*+1, −*y*+1, −*z*+1; (viii) −*x*+1, −*y*+2, −*z*+1.
